# Editorial: Microbiotechnology Based Surfactants and Their Applications

**DOI:** 10.3389/fmicb.2015.01344

**Published:** 2015-12-01

**Authors:** Pattanathu K. S. M. Rahman, Kamaljeet K. Sekhon Randhawa

**Affiliations:** ^1^School of Science and Engineering, Technology Futures Institute, Teesside UniversityMiddlesbrough, UK; ^2^Section for Sustainable Biotechnology, Aalborg UniversityCopenhagen, Denmark

**Keywords:** biosurfactants, bioemulsifiers, actinobacteria, enzymes, market research

This editorial is an annotation on the exciting research topic “Microbiotechnology based surfactants and their applications” that covers a compilation of original research articles, reviews and mini-reviews submitted by researchers enthusiastically working in the field of biosurfactants.

Biosurfactants, which for a long time have been confused with bioemulsifiers, derived their name from biologically produced surfactants. The term “Surfactants” was, however coined by Antara products in 1950—which covered all products having surface activity, including wetting agents, emulsifiers, dispersants, detergents, and foaming agents. The terms biosurfactants and bioemulsifiers have been used interchangeably for a long time until a demarcation has been suggested by several researchers including (Uzoigwe et al., 2015). They emphasized that although biosurfactants and bioemulsifiers are both amphiphilic in nature and produced by variety of microbes, there are marked differences between them in terms of their physico-chemical properties and physiological roles. Authors strongly presented their opinion that bioemulsifiers are not biosurfactants as only biosurfactants have the surfactant effect of reducing surface tension, although both can emulsify solutions. Debating on the topic of emulsification, another study by Das et al. (2014) from China, showed that emulsification potential and also the antimicrobial activity of rhamnolipid biosurfactants produced by crude oil extracted *Pseudomonas* sp. IMP67 is effected by the ratio of monorhamnolipid (MRL) and dirhamnolipid (DRL) congeners. The MRL and DRL congeners were analyzed by thin layer chromatography and rhamnose quantification. Rhamnolipids from *Pseudomonas* sp. IMP67 also reduced the minimum inhibitory concentrations (MICs) of some antibiotics signifying the synergistic role of these rhamnolipids with antibiotics.

If there is one major stumbling block in the flourishing of the business of biosurfactants it is their high cost of production. There are many factors that can play a significant role in order to bring down the expenses and make the process cost-effective. One such factor is the usage of low-cost substrates for the production of biosurfactants. Second to this could be the exploration of new strains or strains and classes which has been less-explored for biosurfactant production. An extensive review by Kugler and co-authors precisely talks about the class *Actinobacteria* and suggest a lack of structural information on a large proportion of Actinobacterial surfactants. Authors claim that the sheer magnitude of Actinobacterial surfactants that still remains undetermined is evident from this comprehensive review (Kügler et al., 2015). A better understanding of the diversity of the Actinobacterial surfactants would allow to further explore their potential for various novel biotechnological applications just as in case of lipopeptide biosurfactants produced by many microorganisms including *Bacillus* species. Lipopeptides, a series of chemical structural analogs of many different families, are one of the five major classes of biosurfactants known. Among the different families identified, 26 families covering about 90 lipopeptide compounds have been reported in last two decades (Liu et al., 2015).

Not only the less-researched strains and classes but a significant leap is required investigating the carbon sources that would work best for high biosurfactant production. Addressing this area are the original research articles by Antoniou et al. (2015), Gudiña et al. (2015) and Ismail et al. (2014), and a review by Banat et al. (2014).

Eleftheria Antoniou and co-researchers from Greece, investigated the biosurfactant production yield of marine hydrocarbon degraders isolated from Elefsina Bay (Eastern Mediterranean Sea) in presence of heavy oil fraction of crude oil as substrate. Their data particularly emphasized on *Paracoccus marcusii* to be an optimal choice for various bioremediation applications. They reported that the isolated pure strains were found to have higher specific production yields (50 ± 20 mg/l) than the complex microbial marine community-consortia (20 mg/l) (Antoniou et al., 2015). Crude oil was the best energy source for these marine hydrocarbon degraders whereas corn steep liquor (CSL) turned out to be an ideal substrate for *Bacillus subtilis* #573 (Gudiña et al., 2015). Authors reported a yield of 1.3 g/l surfactin using 10% CSL in the medium, which increased to as high as 4.8 g/l when supplemented with the optimum concentration of three metals (iron, manganese, and magnesium) simultaneously. Wael Ismail and his team on the other hand came out with another interesting finding that the expression levels of the *rhl*ABC genes in *Pseudomonas* sp. strain AK6U greatly varies depending on the sulfur source. They showed that a biosurfactant yield of 1.3 g/l was obtained in presence of dibenzothiophene (DBT) as a carbon source which was higher than obtained in presence of DBT-sulfone (0.5 g/l) and the inorganic sulfate (0.44 g/l) (Ismail et al., 2014). To bring together these types of “carbon-source” based studies for “low-cost” biosurfactant production technologies Ibrahim M. Banat and co-authors wrote an intensive review where they discussed how and why despite so many developments on biosurfactants their commercialization remain difficult, costly and to a large extent irregular and what role does the low-cost renewable raw substrates and fermentation technology play in reducing the overall production cost.

Some other interesting studies that focus on rhamnolipids and their applications are also included under this special research topic. Madsen et al. (2015), compared the impact of anionic biosurfactant rhamnolipid and the synthetic surfactant SDS on the structure and stability of three different commercially used enzymes—the cellulase Carezyme®, the phospholipase Lecitase Ultra® and the α-amylase Stainzyme® and found a fundamental difference in their mode of action. In another exciting study on rhamnolipids, Silva et al. (2015), evaluated the potential larvicidal, insecticidal, and repellent activities of rhamnolipids and reported their positive effect against *Aedes aegupti* mosquitoes. Wang et al. (2014), for the first time report the complete pathway of the di-rhamnolipid synthesis process in the genus *Dietzia* and provided insights into the biosurfactant production, oil degradation and removal potential of *Dietzia maris* As-13-3.

From a simple idea of growing bacteria and fungi on immiscible substrates and producing surface-active compounds, to a hurl of more than 250 patents filed in close to three decades followed by a market value expected to reach $2,210.5 million by 2018, biosurfactant industry certainly stands on a substantial fundament. Such stimulating facts and figures are broadly discussed in the opinion article by Sekhon Randhawa and Rahman (2014).

Apart from their industrially diverse applications in the field of bioremediation, enhanced oil recovery, cosmetic, food, and medical industries biosurfactants can boast off their unique eco-friendly nature to attract consumers and give the chemical surfactants a tough competition in the global market. The pharmaceutical applications such as biological usage as antiviral, antitumor, antibiotic agents, as insecticides, fungicides, and immune-modulators or enzyme inhibitors have not been fully realized. With the stringent governmental regulations coming into effect in favor of production and usage of the bio-based surfactants, more and more companies are working on the commercialization of the production technology of biosurfactants and to bring down their higher prices. There is no dearth of astonishing applications of biosurfactants; the only challenge is their supply through bio-based production methods to meet the demands well in time.

## Author contributions

PR initiated the research topic and co-ordinated the entire editorial process. There are 11 manuscripts accepted for publication in this research topic contributed by 55 authors from UK, Denmark, Greece, Germany, South Africa, India, Brazil, Bahrain, Portugal, and China. He has initiated peer review process by inviting experts from Germany, Spain, USA, Trinidad and Tobago, India, Denmark, China, Bahrain, Malaysia, Japan, and UK. The reviewers' co-operation and timely responses to complete the research topic is highly commendable. KS was not assigned as Topic Editor for this research topic but she has contributed as Co-author in this Editorial commentary. She is an expert on this topic and has been working specifically in this field, she has her adept inputs in conceptualizing the commentary, analysing the data, interpreting and drafting the commentary. KS has given her final approval of the version to be published and agrees to be held accountable for all aspects of the work related to the accuracy and integrity.

### Conflict of interest statement

The authors declare that the research was conducted in the absence of any commercial or financial relationships that could be construed as a potential conflict of interest.
